# Haemodynamics of Hypertension in Children

**DOI:** 10.1007/s11906-020-01044-2

**Published:** 2020-08-25

**Authors:** Ye Li, Emily Haseler, Phil Chowienczyk, Manish D. Sinha

**Affiliations:** 1 King’s College London British Heart Foundation Centre, London, UK; 2 Department of Clinical Pharmacology, St Thomas’ Hospital, Lambeth Palace Road, London, SE1 7EH UK; 3 Department of Pediatric Nephrology, Evelina London Children’s Hospital, London, UK

**Keywords:** Blood pressure, Cardiac output, Haemodynamics, Hypertension, Arterial stiffness

## Abstract

**Purpose of Review:**

To review the haemodynamic characteristics of paediatric hypertension.

**Recent Findings:**

Pulsatile components of blood pressure are determined by left ventricular dynamics, aortic stiffness, systemic vascular resistance and wave propagation phenomena. Recent studies delineating these factors have identified haemodynamic mechanisms contributing to primary hypertension in children.

**Summary:**

Studies to date suggest a role of cardiac over activity, characterized by increased heart rate and left ventricular ejection, and increased aortic stiffness as the main haemodynamic determinants of primary hypertension in children.

## Introduction

Hypertension in the adult population is the leading cause of cardiovascular morbidity and has been estimated as responsible for one third of ischaemic heart disease and two thirds of stroke [1]. Hypertension in older adults is thought to be primarily due to a degenerative vascular ageing process leading to stiffening of the large arteries and possibly other haemodynamic effects [2]. The aetiology of primary hypertension in children and young people is less well understood. Hypertension in the paediatric population is becoming more prevalent, mainly due to the increasing prevalence of obesity. Longitudinal studies have shown that children with hypertension are likely to become hypertensive adults with an elevated cardiovascular risk [3]. This review aims to summarize what is currently known regarding the haemodynamic mechanisms underlying paediatric hypertension and suggest directions for future research in the area.

## Definition and Prevalence of Hypertension in the Paediatric Population

There is no consensus from longitudinal data regarding what degree of elevation in systolic and diastolic blood pressure is associated with end organ damage and adverse cardiovascular outcomes in children. Paediatric hypertension is therefore diagnosed using a statistical definition of either mean systolic blood pressure (SBP) or mean diastolic blood pressure (DBP), measured on at least 3 separate occasions, above the 95th percentile for height, gender and age of a reference population [4]. The prevalence of hypertension thus varies according to characteristics of the local population and has been found to be between 2.2% - 13% in recent reports [5–9]. Paediatric hypertension may occur secondary to other conditions, including renal, renovascular, oncological and endocrine conditions plus iatrogenic causes [10••]. However, there is an increasing prevalence of primary hypertension in children and adolescents associated with obesity [7, 11, 12•, 13]. One large study reported a prevalence of 22% in a large multinational cohort of overweight and obese children [14]. Another estimated that up to 37% of cases of paediatric hypertension may be secondary to obesity [6].

Isolated systolic hypertension (ISH) is more common than systo diastolic (raised SBP and DBP) hypertension in the elderly adult population, presumed due to age-related stiffening of the large arteries [15]. Recent data show ISH is also the most common hypertensive phenotype in young adults: a prevalence of 8% for ISH and 12% prevalence for total hypertension were reported in the ENIGMA study [16••]. Data in children are more limited but suggest that ISH also predominates over systo diastolic hypertension; one study in 10–14 year olds found their hypertensive cohort to comprise of 80% ISH [6]. Another found ISH to represent 88% of hypertension in 12–16 year old adolescents [17]. These data suggest that different haemodynamic mechanisms may contribute to the development of hypertension across the age spectrum from childhood into middle age and old age.

## Static and Pulsatile Components of BP

Blood pressure (BP) is commonly measured as SBP, the pressure in the artery during ventricular contraction, and DBP, the pressure in the artery when the ventricles are relaxed. A more physiological description of BP is its separation into static and pulsatile components [18–20]. The static or ‘steady state’ component is represented by the mean arterial pressure (MAP). MAP is the perfusion pressure driving blood flow to various organs. As the product of cardiac output (CO) and systemic vascular resistance (SVR, through the definition of SVR as a resistance by analogy to Ohm’s law), an elevated MAP may be due to elevation in CO, SVR or both. This has led to the concept that hypertension could be cardiac related (high CO), vascular related (high SVR) or mixed due to a combination of increased CO and SVR [21].

The main pulsatile component of BP is pulse pressure (PP), defined as the difference between SBP and DBP, and is mainly determined by the interaction of ventricular ejection with the compliance or “buffering-capacity” of the large arteries [20, 22]. In adult hypertension the term “arterial stiffness” has been widely used and this is inversely related to compliance. Depending on left ventricular (LV) ejection dynamics, large artery compliance and SVR, different values of PP may arise for the same value MAP. Since hypertension in children is often ISH characterized by a raised PP, separating BP into its static and pulsatile components may offer better insight into pathophysiology of paediatric hypertension.

## Determinants of the Static Component of BP

### Cardiac Output: Evidence for a Hyperdynamic State in Hypertensive Children?

**Fig. 1:**
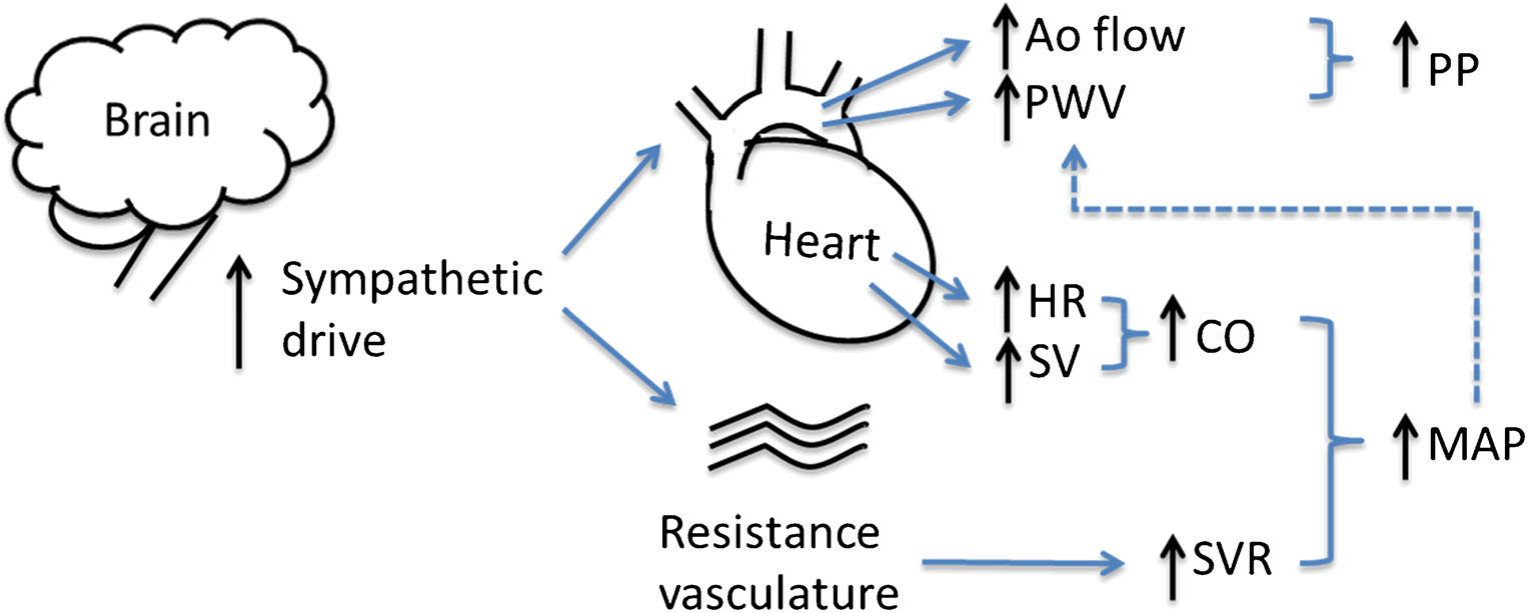
Possible haemodynamic mechanism associated with obesity hypertension in children. Increased early left ventricular ejection and aortic flow velocity (Ao flow) together with increased aortic pulse wave velocity (PWV) may cause an increase in pulse pressure (PP). Increased heart rate (HR) and stroke volume (SV) may lead to an increase in cardiac output (CO) which together with normal or increased systemic vascular resistance leads to an increase in mean arterial pressure (MAP). The increase in PWV may be primary or secondary to pressure-dependence and increased MAP (dashed line). All these mechanism could be caused by an increase in sympathetic drive but many other potential underlying causes (e.g. renal sodium retention) are possible

It has been suggested that in younger adult hypertensive cohorts, a ‘hyperdynamic’ state exists in which heart rate (HR) and/or stroke volume (SV) are increased [23]. In these patients, increased HR and/or SV leads to increased CO, contributing to increased blood pressure and subsequent hypertension (Fig. 1). However, the evidence for this hypothesis is not conclusive; findings in favour [16, 24, 25••] or challenging [26, 27] a hyperdynamic state have been reported. In most studies increased CO has been positively associated with elevated BP [16, 24, 28]. There are also some data supporting gender differences in the contribution of increased cardiac output to hypertension; an analysis from the ENIGMA cohort in young adults showed that males with hypertension tended towards a ‘cardiac’ phenotype, with elevated HR, SV and CO [29]. Females on the other hand tended towards increased SVR and pulse wave velocity (PWV, a measure of arterial stiffness) described as a ‘vascular’ phenotype.

### Relationship between Cardiac Output and Obesity

Most data supporting the contribution of a hyperdynamic phenotype in the aetiology of paediatric hypertension have focused on obese cohorts, or those with a high proportion of obese individuals: data from the Bogalusa Heart study showed that resting HR was positively correlated with both markers of obesity (subscapular skin fold thickness) and BP [30]. Sorof et al. found that HR was highest in obese hypertensive schoolchildren, and lowest in normotensive, non-obese children [17]. Compared with normotensive children, hypertensive children with obesity or overweight have higher CO [31, 32]. This increased CO is thought to be driven by the increased metabolic demands seen in overweight/obese children [33].

An obese individual is expected to have higher SV and CO due to their increased cardiac size compared to a non-obese individual. Increased SV and CO in the context of hypertension may either be physiological as a result of the increased body size or elevated even when adjusted for body size; ‘normalizing’ CO and SV to body size may help to make appropriate comparisons between obese and non-obese individuals [33, 34]. In the ENIGMA study [16••], SV remained significantly elevated in hypertensive young adults even when corrected for body surface area. Chirico et al. found increased HR and CO in hypertensive compared to normotensive children [24]. Although 67% of the hypertensive children were obese, there was no significant association between HR and BMI. In a large population study of 17,000 Chinese children aged 6–12 years, both obesity and HR were positively correlated with risk of hypertension [9]. The association between elevated HR and elevated BP suggests a role for increased sympathetic nervous system activity and a hyperdynamic state in development of primary hypertension [17]. However, whether a hyperdynamic circulation plays a significant role for non- obese children is not clear from the current research.

### Systemic Vascular Resistance

SVR is regulated by small muscular arteries and arterioles [35]. According to the Hagen-Poiseuille equation, resistance (R) of each arteriole is given by: R = 8L.η/(π.r^4^), where L is the length of the vessel, η is the viscosity of blood and r is the radius of the blood vessel. The radius of the small arteries or arterioles thus has a profound effect on the resistance to blood flow and SVR will be determined by this and the density of the microvasculature. Most previous studies have reported no difference in SVR between normotensive and hypertensive adolescents and young adults [16, 24, 25••, 36]. However, this finding was not corroborated by Lund [26] or Julius et al. [23], who observed higher SVR in hypertensive young adults. More recently, data from Avon Longitudinal Study of Parents and Children (ALSCPAC) [27], including 2091 healthy 17 year old participants, suggested that higher BP is attributable to a combination of higher CO and higher SVR.

In a follow-up analysis of the ENIGMA cohort [37], differing relationships were found between CO, SVR and BP according to BMI. SVR was positively correlated with BP in overweight/obese young adults. Conversely, CO was more strongly associated with SBP in lean young adults. This suggests that different mechanisms are likely to contribute to SBP in obese compared to non-obese young people. In addition, lean body mass was found to be a better correlate than fat mass for SV, CO and SVR [38].

## Determinants of the Pulsatile Component of BP

PP differs according to whether it is measured at peripheral sites (e.g. in the brachial artery as is usual) or at a “central” site such as the proximal aorta. However, peripheral and central PP is closely correlated [39]. The water hammer equation states that in early systole, in the absence of wave reflections, central PP (which in children arises early in systole and coincides with the first peak of the aortic BP waveform) is determined by proximal aortic PWV and aortic blood flow velocity (U) through the equation: *PP* = *ρ* × *PWV* × *U*, where ρ is the density of blood. Previous work using a combination of simulated data obtained from computational modelling and experimental data supports this theoretical relationship [40].

### Large Artery Pulse Wave Velocity

Measurement of aortic/large artery PWV and arterial compliance (inversely related to PWV) over various segments of the arterial tree and using various methods is being performed in increasing numbers of paediatric studies. A large study has shown a graded increase in carotid-femoral PWV from normotensive to prehypertensive to hypertensive youth aged 10–23 years [41]. A recent cardiac magnetic resonance study [36] reported lower total arterial compliance and ascending aortic compliance in hypertensive compared to normotensive children. Substantial attention has been given to the relationship of PWV to obesity in children. Children and adolescents with hypertension and BMI in the overweight/obese range present with higher values of carotid-femoral PWV than their peers with normal body weight [42–44]. One previous study has demonstrated a correlation between PWV and continuous metabolic syndrome score in Indian children [45].

Very few studies have addressed the question of whether higher PWV is a cause of or a consequence of elevated MAP. Pressure dependence of PWV due to distension of the arterial wall transferring wall stress to stiffer elements within the wall is well recognized. In a recent study [25••], we observed increased proximal aortic PWV in hypertensive children, which remained significant after statistical adjustment for MAP. However, the difference between proximal PWV in the hypertensive and control groups was 0.8 m/s and that for MAP 14 mmHg giving a ratio of 0.6 m/s per 10 mmHg which could potentially be explained by pressure-dependence. In the ENIGMA study, ratios of 0.3 m/s per 10 mmHg were found [16••]. In this large cohort, most young adults with ISH had either elevated SV, or both elevated SV and PWV. However, there was a subgroup that had normal SV and increased PWV. This may represent a distinct group for whom premature arterial stiffening is the primary haemodynamic phenotype underlying the development of hypertension, whether from premature vascular ageing or other mechanisms yet to be elucidated.

### Left Ventricular Dynamics and Aortic Flow

Whilst most attention has focused on arterial stiffness as a cause of raised PP, the water hammer equation shows that PP may be dependent on aortic flow characteristics. Whilst SV has been measured in many studies aortic flow velocity has been reported in relatively few. Since pulsatile BP components and PWV are linked through aortic flow velocity, aortic flow velocity may also be an important determinant of hypertension and deserves further evaluation. Measuring PWV and flow velocity profiles should allow the pulsatile component, PP, to be partitioned into effects arising purely as a result of aortic stiffening and those that arise through altered ventricular dynamics and hence flow velocity [40]. Ventricular dynamics will be dependent on the intrinsic contractility of the myocardium and afterload imposed by the impedance of the vascular tree (largely determined by proximal PWV and SVR). Rushmer et al. [46] first suggested that peak aortic flow velocity might be useful indicator of left ventricular ejection function. A previous study [47] reported higher peak aortic flow in overweight/obese hypertensive adolescents compared to normotensive controls. A recent study by ourselves [25••] also observed that the peak aortic flow velocity was higher in hypertensive compared to normotensive children, and suggested increased PP was explained both by increased aortic stiffness and increased left ventricular ejection velocity.

### Pressure Wave Reflection

The phenomenon of pressure wave reflection – reflection of a forward propagating pressure wave generated by ventricular contraction from peripheral sites in the arterial tree to form a backward wave that summates with the forward wave is thought to be responsible for the peripheral amplification of pulsatile components of BP. Whether and to what extent reflection contributes to elevated BP is uncertain [48]. One of the problems in studying this phenomenon is obtaining a direct measure of “reflection”. Aortic augmentation index (AIx), the ratio of the increment between the second and first peaks of the aortic BP waveform (which in children may be negative) to central PP has been widely used as an indirect measure of reflection [49]. However, it is now recognized that AIx is influenced by ventricular dynamics [50]. The ENIGMA study [16••] reported AIx to be significantly lower in young adults with ISH, compared to control group. Other studies [25••, 32, 51••] have reported no significant difference in AIx between hypertensive and control children and adolescents. A more direct measure of reflection is the reflection coefficient, obtained by mathematical separation of forward and backward waves (from pressure and flow), as the ratio of the amplitude of the backward to forward wave. In two of these studies [25••, 51••], reflection coefficient was calculated. Both studies found that forward and backward components were higher in hypertensive children but the backward pressure was likely secondary to the increased forward wave as the reflection coefficients were similar in hypertensive and control groups.

## Increased Cardiac Output as a Precursor to Hypertension in Children

There is some evidence that cardiac markers of sympathetic activity may feature in patients before the development of peripherally detectable hypertension. Palatini studied 163 young adult patients with grade 1 hypertension and otherwise low cardiovascular risk. HR variability at baseline was used as a proxy for sympathetic activity. Patients with decreased HR variability were found to be at increased risk of development of sustained hypertension at follow-up. These patients also had increased HR at baseline and decreased large and small artery compliance at follow-up [52]. Totaro et al. compared peripheral and central BP and found that young adults who were normotensive on peripheral measurement but had high central BP had higher HR with signs of target organ damage including increased carotid intima-media thickness (cIMT) and left ventricular mass [53]. One of the few studies in children in this area also used HR variability as a marker of sympathetic nervous system activity and found decreased HR variability was associated with risk of being hypertensive or pre-hypertensive, after adjusting for adiposity [54].

## Relationship between Blood Pressure in Childhood and Adverse Cardiovascular Outcomes

Blood pressure trajectories appear to track from childhood into adulthood and throughout adult life [55], a tendency that may be stronger in males [56, 57]. There is also some evidence that hypertension in children can be tracked back to prehypertensive and white coat hypertension states: for example, in one study with two ambulatory BP (ABPM) readings a minimum of 6 months apart, BP increased in 47%, with 18 and 19% of the children with normal and pre-hypertensive profiles respectively going on to develop hypertension at follow-up [58]. An earlier large cohort study estimated a 7% rate of progression per year from a prehypertensive to a hypertensive state [59].

With regard to whether childhood hypertension is related to cardiovascular morbidity as an adult, data are more scarce. Hypertension as an adolescent has been shown to be associated with a threefold increase in mortality from stroke in midlife [60] and associated with an increased likelihood of coronary artery disease in later life, albeit in a small sample [61]. A large cohort study found that persistently raised BP through childhood to adulthood was associated with a higher cIMT as an adult, whereas if hypertension had resolved by adult years this risk was reduced [62].

A large multicentre cohort study is currently ongoing to address this gap in knowledge: the International Childhood Cardiovascular Cohort Consortium Outcomes Study (i3C Outcomes), which thus far has managed to recruit 10,968 participants from 7 international cohorts recruited over the last 50 years [63•]. This study aims to better delineate the relationships between cardiovascular risk factors in childhood and adult cardiovascular endpoints. In addition, a large prospective cohort study, the Study of High Blood Pressure in Paediatrics: Adult Hypertension Onset in Youth (SHIP-AHOY) is currently underway and aiming to recruit 500 adolescents. Participants will undergo a number of haemodynamic, biochemical and genetic assessment in order to investigate whether intermediate markers of target organ damage can be used to move from the current statistical definition of hypertension towards one based on risk of morbidity [64•]. These studies will help in the management of paediatric hypertension, particularly with treatment thresholds and targets. However, the tracking of BP from childhood to adulthood and the established risk associated with adult hypertension is sufficiently strong for elevated BP in children to be regarded as one of the most important conditions influencing population health.

## Implications of the Haemodynamics Characteristics of Paediatric Hypertension and Future Research

As has been reviewed above, many of the mechanisms contributing to hypertension in children involve some kind of cardiac “over-activity”: increased HR, increased early systolic LV ejection with increased flow velocity and increased SV. Conversely SVR, which is thought to be important in adult hypertension, may be less important in childhood hypertension. Given the tracking of BP from childhood into adulthood, this has implications for the aetiology of adult hypertension. It may be that increased SVR develops as a remodelling process to normalize arterial wall stress and that there is a transition from a predominantly “cardiac” phenotype to a “vascular” phenotype. Longitudinal studies with detailed haemodynamic phenotyping will be required to test this hypothesis.

From a practical perspective, a cardiac form of hypertension in childhood is likely to benefit from specific interventions to decrease sympathetic activity and cardiac drive. Preventing or reversing weight gain is an obvious intervention but other interventions that reduce cardiac drive could be beneficial. In particular, beta-adrenergic blockade, which is thought to be less effective that other forms of antihypertensive treatment in adults, might be more effective in children. Clinical trials comparing the efficacy of different antihypertensive treatments with stratification by haemodynamic phenotype will be required to test this hypothesis.

Increased aortic/large artery stiffness is a common finding in paediatric hypertension. A key question with implications both for the aetiology and treatment of paediatric hypertension is whether this occurs secondary to an increase in BP due to other (e.g. cardiac) causes or is a primary cause. Again, longitudinal studies to investigate whether PWV normalizes in parallel with BP reduction and interventional studies will be required to determine the importance of arterial stiffness.

## Conclusions

This topical review has addressed recent investigations related to the haemodynamic mechanism of hypertension in children. The review has expanded on the determinants of the static and pulsatile components of blood pressure. Abnormalities in function of the left ventricle and large arteries, and their interaction have been neglected in paediatric hypertension research until recently, but may be of critical importance in understanding the development and consequences of paediatric hypertension. There is convincing evidence of a substantial “cardiac” and large artery component to paediatric hypertension that has implications for the aetiology of both paediatric and adult hypertension and for the prevention and treatment of hypertension. Future longitudinal studies and clinical trials are required to definitively address the benefits of targeting treatment to the haemodynamic phenotype.
